# Detoxification enzymes associated with insecticide resistance in laboratory strains of *Anopheles arabiensis* of different geographic origin

**DOI:** 10.1186/1756-3305-5-113

**Published:** 2012-06-07

**Authors:** Luisa Nardini, Riann N Christian, Nanette Coetzer, Hilary Ranson, Maureen Coetzee, Lizette L Koekemoer

**Affiliations:** 1 Vector Control Reference Unit, Centre for Opportunistic, Tropical and Hospital Infections, National Institute for Communicable Diseases of the National Health Laboratory Services, Private Bag X4, Sandringham, 2131, Johannesburg, South Africa; 2 Malaria Entomology Research Unit, School of Pathology, Faculty of Health Sciences, University of the Witwatersrand, Johannesburg, South Africa; 3 Bioinformatics and Computational Biology Unit, Department of Biochemistry, University of Pretoria, Pretoria, South Africa; 4 Vector Research Group, Liverpool School of Tropical Medicine, Liverpool, UK

**Keywords:** *Anopheles arabiensis*, Insecticide resistance, Microarrays, Detoxification enzymes, *kdr*

## Abstract

**Background:**

The use of insecticides to control malaria vectors is essential to reduce the prevalence of malaria and as a result, the development of insecticide resistance in vector populations is of major concern. *Anopheles arabiensis* is one of the main African malaria vectors and insecticide resistance in this species has been reported in a number of countries. The aim of this study was to investigate the detoxification enzymes that are involved in *An. arabiensis* resistance to DDT and pyrethroids.

**Methods:**

The detoxification enzyme profiles were compared between two DDT selected, insecticide resistant strains of *An. arabiensis,* one from South Africa and one from Sudan, using the *An. gambiae* detoxification chip, a boutique microarray based on the major classes of enzymes associated with metabolism and detoxification of insecticides. Synergist assays were performed in order to clarify the roles of over-transcribed detoxification genes in the observed *resistance* phenotypes. In addition, the presence of kdr mutations in the colonies under investigation was determined.

**Results:**

The microarray data identifies several genes over-transcribed in the insecticide selected South African strain, while in the Sudanese population, only one gene, *CYP9L1*, was found to be over-transcribed. The outcome of the synergist experiments indicate that the over-transcription of detoxification enzymes is linked to deltamethrin resistance, while DDT and permethrin resistance are mainly associated with the presence of the L1014F *kdr* mutation.

**Conclusions:**

These data emphasise the complexity associated with resistance phenotypes and suggest that specific insecticide resistance mechanisms cannot be extrapolated to different vector populations of the same species.

## Background

In 2009, the World Health Organization (WHO) estimated 225 million cases of malaria worldwide [1]. Of these, 800 000 cases resulted in death, and most of these deaths occurred in Africa where infants, young children and pregnant women were, and still are, worst affected [1]. Insecticide use has been the most successful way of controlling malaria vectors, and as such, controlling the disease. As a result, the development of insecticide resistance in vector populations has had a major impact on malaria transmission and control.

*Anopheles arabiensis* is one of the major African malaria vectors and belongs to the *An. gambiae* complex. Resistance in this species has been reported in a number of countries and to a range of insecticides. Examples include dichlorodiphenyltrichloroethane (DDT), deltamethrin and permethrin resistance in Ethiopia [2,3]; partial resistance to permethrin in Tanzania [4]; DDT, permethrin, malathion and bendiocarb resistance in Sudan [5,6]; DDT and permethrin resistance in South Africa [7,8]; and resistance to propoxur in Mozambique [9].

Insecticide resistance is either based on an increase in levels of detoxification enzymes [10], or is related to reduced target-site sensitivity [10,11]. Detoxification enzymes that are associated with insecticide resistance belong to large enzyme families, known as super-families. In *An. gambiae* there are multiple cytochrome P450s (n = 111) [12-16], esterases (n = 51) [14,15,17] and 31 glutathione S-transferase (GSTs) genes [14,15,17]. Numerous genes form part of these families and for this reason, it is difficult to determine the specific gene(s) associated with resistance to a particular insecticide, or class of insecticides. The development of high throughput technology such as microarrays provided a solution to this problem [18]. The *An. gambiae* detoxification microarray is a custom–made boutique array that includes GSTs, esterases and P450s as well as number of redox genes that are associated with P450 metabolism and which protect against free radical damage [19].

In addition to detoxification enzyme mediated protection against insecticides, a number of target-site resistance mechanisms are known. One of the best studied mechanisms in *An. gambiae* is *‘kdr’* or knockdown resistance. This mechanism is characterized by a mutation in the voltage gated sodium channel that confers resistance to both DDT and pyrethroids [2,20-25]. However, the relationship between *kdr* and cross resistance between insecticide classes is not as clear cut as previously assumed [26]. In *An. gambiae* for example, the presence of *kdr* is most strongly correlated with DDT resistance, followed by permethrin resistance, while the weakest association is with the deltamethrin resistant phenotype [27].

In the original study in which the ‘detox chip’ was presented, the expression profile of detoxification genes associated with DDT resistance in a laboratory colony of *An. gambiae* was determined [19]. Genes that were over-transcribed included *GSTE2,* a gene that has previously been linked to DDT resistance [28,29], as well as *CYP6Z1,PX13A,PX13B* and *CYP12F1.* Since then, the detox chip has been used in several other studies. More recently, permethrin resistance in a wild *An. gambiae* population was monitored using the detox chip [30]. Three P450s showed high expression levels: *CYP6P3,CYP4H24* and *CYP4H19.* Although the detox chip was constructed using *An. gambiae* sequence information, it has been used with success in a number of cross-species hybridizations with *An. arabiensis*[31], *An. funestus*[32] and *An. stephensi*[33].

The aim of this study was to compare the transcription of detoxification enzymes of two laboratory strains of insecticide-resistant *An. arabiensis.* The colonies were originally derived from different geographic locations, one from Sudan and the other from South Africa. In Sudan, vector control includes the use of long lasting insecticide-treated bed nets (LLINs), temephos for larviciding, and bendiocarb is used for IRS [34]. South African vector control approaches include the use of IRS with DDT in traditional unplastered mud, grass or wooden houses and pyrethroids on walls with enamel painted surfaces [35,36].

## Methods

### Mosquito colonies

Mosquitoes were maintained under standard insectary conditions of 26 ± 2°C, a relative humidity of 70-80%, with a 12:12 light:dark cycle and 45 minute dusk/dawn period. The strains used for this study were as follows: *An. arabiensis,* colonized in the 1980’s from the Sennar region of Sudan (SENN) and *An. arabiensis,* colonized in 2002 from the KwaZulu-Natal (KZN) province in South Africa (MBN). For each colony, both a susceptible or “unselected” strain (called the “base colony”) was available, as well as a DDT-resistant strain. The resistant strains have been under continuous DDT selection from the time of colonization. To maintain resistance in the selected colonies, three day old adults were exposed to 4% DDT in every generation using World Health Organization (WHO) insecticide tubes and procedures [37]. Both DDT selected strains from Sudan and South Africa showed very low or no mortality (after 24 hr recovery period), following exposure to DDT for 1 hr and both were homozygous for the L1014F *kdr* mutation, as confirmed by PCR using AGD1 and AGD2 primers [23], and sequencing in both directions (data not shown). All strains are maintained in separate insectary rooms to minimise the chance of contamination between strains.

### World Health Organization insecticide susceptibility assays

The insecticide resistance status of the colonies were evaluated against a range of insecticides including DDT (4.0%), permethrin (0.75%), deltamethrin (0.05%), bendiocarb (0.1%), propoxur (0.1%) and fenitrothion (1.0%). The assays were done in order to confirm the resistance status of each strain. Assays were performed according to standard WHO procedures [37].

### Synergist assays

Piperonyl butoxide (PBO), an inhibitor of monooxygenase activity, and diethyl maleate (DEM), an inhibitor of GSTs, were used to synergise the resistant colonies, SENN-DDT and MBN-DDT. Twenty-five 2 to 3 day old mosquitoes were exposed to 4.0% PBO (SENN-DDT and MBN-DDT) or 8.0% DEM (MBN-DDT) for an hour, and then immediately exposed to insecticide (0.05% permethrin, 0.75% deltamethrin or 4% DDT) for an hour before being returned to a holding tube. In addition, mosquitoes (n ≈ 25) were exposed to the insecticide only (deltamethrin, permethrin or DDT) for an hour, and then as an additional control, to the synergist only (PBO or DEM) for an hour, and were then returned to holding tubes. Mortality was recorded after 24 hours. Insecticide exposure versus synergist plus insecticide exposure were analysed using a *t*-test. Three to four repeats were prepared for each insecticide/synergist assay, depending on mosquito availability.

### RNA extractions and cDNA synthesis for microarrays

Female mosquitoes from the different colonies (SENN-base [susceptible]; SENN-DDT [resistant]; MBN-base [susceptible]; MBN-DDT [resistant]) were collected on the day of emergence and maintained on 10% sugar water. Three days later, RNA was extracted from 15 mosquitoes, representing one biological repeat. A total of three biological repeats were used in the experiment and analysis described below. RNA was extracted as described by Christian *et al*. [32].

### Microarrays

Three independent biological repeats were performed for each colony group (SENN and MBN), and for each biological repeat, two technical repeats were performed that included dye swaps in order to compensate for dye bias. Preparation of the probes and microarrays was based on the protocol of Christian *et al*. [32], with some minor modifications based on the outcome of preliminary experiments. Briefly, amplified antisense (a) RNA was labeled by reverse transcription using Cy-dUTPs. aRNA (8 μg) was mixed with random hexamers (Invitrogen), 2 μl spike in control (Lucidea Universal ScoreCard, Amersham) and water and the mixture was incubated at 70°C for 5 minutes. The reverse transcription mix (RT Buffer, DTT, Cy3-dUTP or Cy5-dUTP, DTT, dT-NTP mix, RNAsin and Superscript^®^ III [Invitrogen]) was added to each RNA and primer mix, and incubated at 50°C for 2.5 hours. The reaction was stopped by adding 1 M NaOH/20 mM EDTA, and incubation at 70°C for 5 minutes. The Cy-labeled cDNAs were purified using the CyScribe™ GFX™ Purification Kit (Amersham) according to manufacturer’s instructions. In order to control the efficiency of the labeling and purification procedures, samples were measured on a NanoDrop using the microarray setting. Acceptable dye binding was considered to be >0.1 pmol/μl and acceptable cDNA yields were required to be >15 ng/μl. If these conditions were not met, the hybridization process was abandoned. Poly(A) was added to each cDNA mix and samples were evaporated at 37°C for an hour using an Eppendorf concentrator 5350. The cDNA was resuspended in 15.5 μl hybridization buffer (Corning) and kept in the dark until slides were ready.

During this time, the microarrays were prepared for hybridization. The Pronto!™ Universal Microarray Hybridization Kit (Corning) was used, but a 1.5x preparation of each wash solution was used, along with slightly reduced exposure times, following a series of optimization experiments. Once slides were prepared, the labeled targets were denatured by hybridization at 95°C for 5 minutes. The targets were added to each array and hybridizations were performed at 42°C for 18–20 hours. After incubation, slides were washed using the Pronto!™ Universal Hybridization Kit (1.5x solutions prepared), and dried by centrifugation at 2500 x *g* for 2 minutes.

### Microarray scanning and data analysis

Analyses were based on those used by Christian *et al*. [32]. The arrays were scanned using the Genepix 4000B scanner (Molecular Devices, USA) where the PMT values were adjusted to give a pixel ratio of approximately 1. Spot quality and background intensities were examined and corrected using Genepix Pro 6.0 software (Axon Instruments, USA). Saturated features were recorded as such, and were excluded from analysis.

Gene expression data were analaysed using Limma version 2.12.0 (Bioconductor) [38] in R, version 2.8.0 (http://cran.r-project.org/bin/windows/base/old/2.8.0/), a command-driven program for statistical computing. Raw intensity values for each spot were calculated, and then background corrected by the method “normexp” with an offset of 50. This approach produces positive adjusted intensities and variation in log-ratios for low intensity spots are pushed toward zero (i.e. no spots are “lost” if a high background signal is measured). The corrected intensity values were transformed to log-ratios and then normalized. Control spots were used for within array normalization (i.e. normalization was based on non-differentially expressed control spots). Between array normalization was done using the “Aquantile” method where spot intensity values are transformed so that their distributions are similar between microarrays. MA-plots were viewed so that normalization could be monitored. Once analyses are complete, Limma produces a “topTable”, a summary that includes the following: the gene ID, M (log_2_-fold change) and A (log_2_-average intensity) values, a moderated *t*-statistic, a *p*-value, an adjusted *p*- value, a B-statistic as well as an F-statistic (from the ‘eBayes’ function). Of interest to us were genes with adjusted p-values ≤ 0.05 and fold-changes ≥ 1.5. Genes in this category were considered to be statistically significant. These data have been deposited into Vectorbase (https://www.vectorbase.org).

### Quantitative real-time PCR (qPCR)

Real-time PCR was carried out in order to validate the results of the microarray experiments. As with the microarray experiment, RNA was extracted from three day old *An. arabiensis* females that had been supplied with 10% sugar solution. RNA was extracted from 15 mosquitoes (one biological repeat) using the TRI-Reagent® Solution (Sigma-Aldrich) and supplied methodology. A DNase treatment was included (RNase-Free DNase Set, Qiagen). Samples were quantified using a NanoDrop and then reverse transcribed into cDNA using the QuantiTect® Reverse Transcription Kit (Qiagen).

cDNA was stored at −20°C until required for PCR. For the SENN colony group, three genes were evaluated by real-time PCR (*CYP9L1* [over-transcribed], *CO1* [saturated] and *CYP4G16* [saturated]), and for the MBN group, four genes were evaluated (*CYP6P3,CYP6AK1,CYP6M2* and *TPX4,* all found to be over-transcribed in the microarray study). Primers were designed based on *An. gambiae* sequence information using either Beacon Designer™ (Premier Biosoft) or Invitrogen’s free online primer design tool, OligoPerfect™ Designer. For each colony, a reference gene evaluation was conducted and the most suitable reference gene was selected from all potential candidate genes tested (ribosomal protein *S7,* ribosomal protein L19 [RPL19], the cytoskeletal protein *β-actin,GAPDH* and TATA binding protein). The data from these experiments were analysed using NormFinder (2004, Molecular Diagnostic Laboratory, Aarhus University Hospital). For the SENN colony group, gene expression was measured relative to *rsp 7,* and for the MBN colony group, gene expression was measured against *β-actin*. PCR was carried out using the Bio-Rad CFX96™ Real-Time PCR Detection System. Each reaction was set up using a total volume of 25 μl comprising 12.5 μl IQ™ SYBR super-mix (Bio-Rad), 4 μl primer (concentration optimised for each gene), 1 μl cDNA (100 ng/μl) and nuclease free water. Primer specifics, including annealing conditions and primer concentrations are described in Table 1 (SENN) and Table 2 (MBN). Standard curves were prepared by two-fold dilutions of cDNA derived from the resistant colony. Three biological repeats were evaluated, and for each biological repeat, three technical repeats were included for each reaction of interest i.e. where relative quantification was calculated. Data were analysed using the Pfaffl [39] method. Initially, PCR product for each gene of interest was sent to Macrogen for sequencing in both directions in order to confirm (over and above melt curve analysis) that the correct product was amplified in each case.

**Table 1:** SENN-base/SENN-DDT primer information for qPCR (F = forward, R = reverse)

**Gene**	**Primer sequence**	**Annealing temperature**	**Amplicon length**
*CYPL9L1*	F 5’- AGA TAA TGT ATT CTT TCG CTA TGG -3’	58.3°C	188
	R 5’- GCT CTT CTC GCT CTT GAA C -3’		
*CO1*	F 5’- TGC TCC TAA AAT AGA AGA AAT TCC -3’	58.3°C	173
	R 5’- TGC TTC CTC CTT CAT TAA CAC -3’		
*CYP4G16*	F 5’- CAG ACC GTC CAG CCA CAT TC -3’	58.3°C	108
	R 5’-GCG AAC GAG CAA TTA TAG GTA CTG -3’		
*rsp 7*	F 5’-TTA CTG CTG TGT ACG ATG CC-3’	58.3°C	135
	R 5’-GAT GGT GGT CTG CTG GTT-3’		

**Table 2:** MBN-base/MBN-DDT primer information for qPCR (F = forward, R = reverse)

**Gene**	**Primer sequence**	**Annealing temperature**	**Amplicon length**
*CYP6M2*	F 5’- CAT GAC ACA AAC CGA CAA GG -3’	60.0°C	235
	R 5’- GGT GAG GAG AGT CGA CGA AG -3’		
*CYP6AK1*	F 5’- TCA TCG AGC GAC AGT GTA CC -3’	58.3°C	251
	R 5’- AAA GTG TGA CCC CAG ACA GG -3’		
*CYP6P3*	F 5’- CGA TTC TTC CTG GAC ATC GT -3’	58.3°C	141
	R 5’- CTT GCC CAA ACT ACC GTC AT -3’		
*TPX4*	F 5’- CAG CTG ACA GAC CGA TTA AG -3’	58.3°C	116
	R 5’- CCG TTC GGG AAC AGT TTG TCT -3’		
*β-actin*	F 5’- ACC AAG AGC CTG AAG CAC -3’	*	123
	R 5’- CGA GCA CGA CAC ACT ATA TAC -3’		

* Annealing temperature used was the same as the target gene of interest.

## Results

### WHO insecticide susceptibility assays

SENN-base (Table 3) was found to be resistant to permethrin (53% mortality), but susceptible to all other insecticides tested. SENN-DDT (Table 3) was resistant to DDT, propoxur, permethrin and deltamethrin, and susceptible to bendiocarb and fenitrothion. MBN-base was susceptible to all insecticides tested, while MBN-DDT was resistant to all insecticides except fenitrothion (Table 3).

**Table 3:** **Mortality data obtained following exposure of (A) SENN-base and SENN-DDT and (B) MBN-base and MBN-DDT to a range of insecticides, all of which belong to classes currently approved by WHO for use in vector control (*n*** **= number of mosquitoes exposed to insecticide)**

**Insecticide**	**SENN-base**		**SENN-DDT**		**MBN-Base**		**MBN-DDT**	
	*n*	*% mortality*	*n*	*% mortality*	*n*	*% mortality*	*n*	*% mortality*
DDT (4.0%)	100	100	99	7.8	88	91.5	96	0
Permethrin (0.75%)	112	53.3	99	7.0	89	97.8	93	4
Deltamethrin (0.05%)	106	99.0	94	50.5	92	100	103	34
Bendiocarb (0.1%)	107	97.8	97	100	95	95.8	102	77.5
Propoxur (0.1%)	89	100	112	85.5	77	100	95	65.3
Fenitrothion (1.0%)	105	100	106	100	94	100	71	100

### Microarrays and qPCR

The *An. gambiae* detox microarray was used in a cross-species hybridization study with *An. arabiensis.* As a result, a subset of arrays used for analysis were checked for probe binding success, (this was a visual assessment) and where probes did not hybridize, the probe name, and its position on the array were recorded. On average, 97.5% binding success rate was obtained in this study.

**Figure 1:**
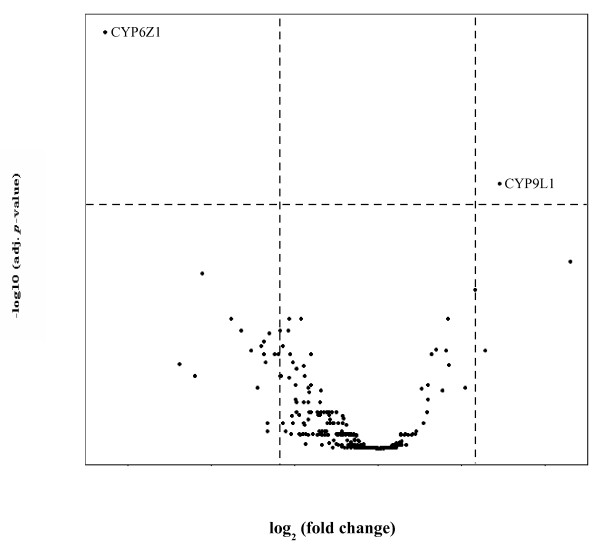
**The volcano plot of SENN-base and SENN-DDT microarray data.** The plot represents both statistical relevance, in the form of the *p*-value on the *y*-axis, and biological relevance in the form of the fold change on the *x*-axis. The cut-offs for significance are shown (adj. *p*-value ≤ 0.05; FC ≥ 1.5) and those genes that meet the criteria are labeled. Note that all positive fold change (FC) values represent genes over-transcribed in the resistant colony (SENN-DDT), while negative FC values represent the genes over transcribed in the susceptible colony (SENN-base).

**Figure 2:**
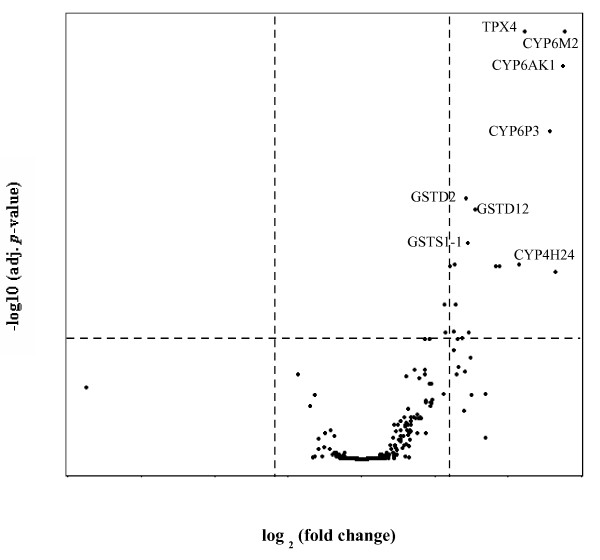
**The volcano plot of MBN-base and MBN-DDT microarray data.** The plot represents both statistical relevance, in the form of the *p*-value on the *y*-axis, and biological relevance in the form of the fold change on the *x*-axis. The cut-offs for significance are shown (adj. *p*-value ≤ 0.05; FC ≥ 1.5) and the top eight genes that met the criteria have been labeled. Note that all positive FC values belong to the genes that are over-transcribed in the resistant colony (MBN-DDT), while negative FC values represent those of the susceptible colony (MBN-base).

Genes that produced a fold change of ≥1.5 and an adjusted *p*-value of ≤ 0.05 after microarray analysis were considered to be differentially regulated. When SENN-DDT was compared with the relevant base colony in the microarray study, only one gene, *CYP9L1,* was found to be significantly over-transcribed (Figure 1). In the unselected equivalent, a single gene, *CYP6Z1,* was over-transcribed. In contrast, in the MBN-DDT colony 20 genes were significantly over-transcribed (Figure 2). Of these, the majority were P450 genes (50%), followed by GSTs (40%) and a small number or redox genes (one TPX and one SOD) (Table 4). Five genes consistently produced saturation on both SENN and MBN microarrays. These were *CYP4G16,CO1,GSTD5,SOD3A* and *AGM1.* The transcription of two of these genes was investigated further by real-time PCR. These genes were assessed using the colonies from Sudan.

**Table 4:** List of probes that were over-transcribed in SENN-DDT and MBN-DDT when compared with the susceptible equivalent

**Gene SENN**	**Function**	**FC**	**Adj.*p*-value**	**GB accession number**	**Location**
*CYP9L1*	Cytochrome P450	1.7	3,74E-2	AF487781	3 L
**MBN**					
*CYP6M2*	Cytochrome P450 monooxygenase	2.7	6.12E-6	AY193729	3R
*TPX4*	Thioredoxin-dependent peroxidase	2.3	6.12E-6	AY745235	3 L
*CYP6AK1*	Cytochrome P450 monooxygenase	2.6	2.12E-5	AY745227	3 L
*CYP6P3*	Cytochrome P450 monooxygenase	2.6	1.20E-4	AF487534	2R
*GSTD2*	Glutathione S-transferase	1.7	3.09E-4	Z71480	2R
*GSTS1-1*	Glutathione S-transferase	1.7	7.49E-4	L07880	3 L
*GSTD12*	Glutathione S-transferase	1.7	1.44E-3	AF316638	2R
*CYP4H24*	Cytochrome P450 monooxygenase	2.2	4.83E-3	AY062206	X
*GSTD3*	Glutathione S-transferase	2.0	4.91E-3	AF513638	2R
*CYP6AG2*	Cytochrome P450 monooxygenase	2.0	5.24E-3	AY745224	2R
*GSTMS3*	Glutathione S-transferase	1.6	5.83E-3	AY278448	3R
*GSTS1-2*	Glutathione S-transferase	1.5	6.71E-3	AF513639	3 L
*CYP9J5*	Cytochrome P450 monooxygenase	2.7	7.73E-3	AY748830	3 L
*CYP6P1*	Cytochrome P450 monooxygenase	1.5	7.73E-3	AY028785	2R
*SOD1*	Superoxide dismutase	1.6	1.13E-2	AY505417	3 L
*CYP6M3*	Cytochrome P450 monooxygenase	1.8	1.58E-2	AY193730	3R
*GSTU1*	Glutathione S-transferase	1.6	1.58E-2	AF515521	X
*CYP12f2*	Cytochrome P450	1.7	1.83E-2	AY176050	3R
*GSTMS1*	Glutathione S-transferase	1.6	3.81E-2	AY278446	X
*CYP12F4*	Cytochrome P450 monooxygenase	1.7	4.01E-2	AY176048	3R

Relevant information included is the gene function, FC, adjusted *p*-value, Genbank (GB) accession number and the chromosomal location of each gene in the *An. gambiae* genome.

**Figure 3:**
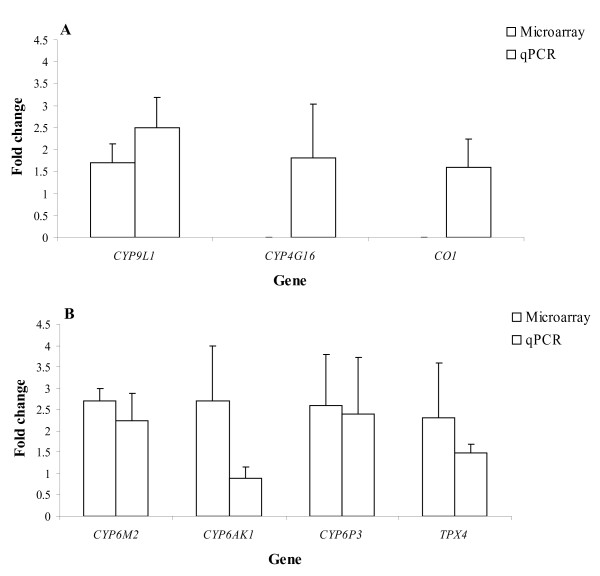
**A comparison of the outcome of gene expression evaluation (mean ± SD) by microarrays and by qPCR in selected genes in (A) the SENN colony group and (B) the MBN colony group.** Genes of interest were measured against the relevant reference genes.

Relative quantification was used to validate the microarray data. The expression level of *CYP9L1* in the Sudanese colony, had a fold change (FC) of 1.7 after microarray analysis, and FC of 2.5 after qPCR analysis (Figure 3A). While saturated spots were flagged and not used in analyses, qPCR was also used to measure the FC difference between the susceptible and resistant Sudanese strains. In two of the five genes that were found to be saturated, the cytochrome oxidase, *CYP4G16,* produced a FC of 1.8, while *CO1,* a gene frequently associated with the resistant phenotype, produced a FC of 1.6 (Figure 3A).

In the case of South African *An. arabiensis* colony (MBN), a sample of four genes that were over-transcribed according to microarary evaluation were validated by qPCR. These genes were the four top genes based on FC and the adjusted *p*-value, namely *CYP6M2,TPX4,CYP6AK1* and *CYP6P3* (Table 4). The FCs in expression after microarray analyses were comparable to those measured by qPCR. Based on qPCR analysis, *CYP6M2* and *CYP6P3* each had fold change expression levels of more than 2 while *CYP6AK1* and *TPX4* produced fold change values of 0.9 and 1.5 respectively (Figure 3B).

### Synergist assays

The synergist assays were used to determine whether the expression of detoxification genes in each colony were in fact related to the resistance observed, or whether the phenotypes were due to the presence of *kdr.* Only one gene (a P450) was over-transcribed in SENN-DDT and so only PBO was used as a synergist in this instance. No significant difference in mortality between DDT exposure versus exposure to PBO + DDT was found (Table 5). Similarly, no significant difference between permethrin versus PBO + permethrin was observed. However, the mortality on deltamethrin versus PBO + deltamethrin was significantly different (*p* = 0.0006, *t* = 7.7308, df = 5) (Table 5). The effects of both DEM and PBO were evaluated in the MBN-DDT colony as monooxygenases and GSTs were over-transcribed in the resistant phenotype according to the microarray experiments. The synergist, PBO, had no significant impact on mosquito response to DDT or permethrin but did impact significantly on MBN-DDT response to deltamethrin (*p* = 0.0004, *t* = 8.331, df = 5). While DEM had no significant impact on DDT and permethrin resistance, a significant difference on mosquito response to deltamethrin versus DEM + deltamethrin (*p* = 0.0083, *t* = 4.8596, df = 4)) (Table 5) was observed.

**Table 5:** **Percentage mortality of SENN-DDT and MBN-DDT mosquitoes (females and males) to DDT and deltamethrin following exposure to synergists (*n*** **= number of mosquitoes tested)**

**Colony**	**Treatment**	***n***	**% Mortality (± SD)**
**SENN-DDT**	PBO (4%) + DDT (4%)	107	3.9 (± 4.7)
	DDT (4%) only	107	13.0 (± 8.6)
	PBO (4%) only	80	0
	PBO (4%) + deltamethrin (0.05%)	126	83.8 (± 1.3)*
	Deltamethrin (0.05%) only	89	25.3 (± 15.6)*
	PBO (4%) only	80	0
	PBO (4%) + permethrin (0.75%)	75	0
	Permethrin (0.75%) only	72	1.3 (± 2.3)
	PBO (4%) only	79	2.7 (± 4.6)
**MBN-DDT**	PBO (4%) + DDT (4%)	79	2.3 (± 2.1)
	DDT (4%) only	71	1.3 (± 2.3)
	PBO (4%) only	81	1.1 (± 2.0)
	PBO (4%) + deltamethrin (0.05%)	78	70.3 (± 16.5)*
	Deltamethrin (0.05%) only	97	2.2 (± 4.3)*
	PBO (4%) only	81	1.1 (± 2.0)
	PBO (4%) + permethrin (0.75%)	74	1.3 (± 2.3)
	Permethrin (0.75%) only	73	6.7 (± 4.6)
	PBO (4%) only	74	1.3 (± 2.3)
**MBN-DDT**	DEM (7%) + DDT (4%)	80	1.5 (± 2.6)
	DDT (4%) only	72	4.0 (± 4.0)
	DEM (7%) only	82	3.5 (± 3.7)
	DEM (7%) + deltamethrin (0.05%)	74	46.0 (± 1.7)*
	Deltamethrin (0.05%) only	78	16.8 (± 10.2)*
	DEM (7%) only	82	3.5 (± 3.7)
	DEM (7%) + permethrin (0.75%)	75	1.7 (± 2.9)
	Permethrin (0.75%) only	75	0
	DEM (7%) only	69	3.0 (± 2.7)

* Indicates significant difference between insecticide versus synergist and insecticide.

## Discussion

Resistance to DDT and pyrethroids is widespread and has hampered malaria control efforts throughout Africa [2-9]. Artificial insecticide resistance selection on laboratory colonies is useful as it allows one to study the resistance mechanism on a population not influenced by other environmental selection pressures. Furthermore, artificial selection in the laboratory allows us to mimic the development of insecticide resistance from repeated and continuous exposure to insecticides, a situation that wild vector populations are frequently exposed to.

The two resistant *An. arabiensis* colonies used in this study, one from South Africa and the other from Sudan, have been under DDT selection pressure in the laboratory. Bioassay data confirmed that both SENN-DDT and MBN-DDT are highly resistant to DDT. In addition to a high level of DDT resistance, the two colonies were found to be resistant to pyrethroids (deltamethrin and permethrin). The South African population showed additional resistance to carbamates, which was not present in the Sudanese colony.

The development of multiple insecticide resistance in the above mentioned colonies is supported by subsequent studies published on the same laboratory populations. The MBN colony was colonized in 2002 without detecting pyrethroid resistance in the population. However, three years later Mouatcho *et al*. [8] reported the presence of pyrethroid resistance, which was rapidly selected for (within four generations) in the laboratory and has been shown to be P450 based. The same author also showed that carbamate tolerance could be selected for from the same colonized field population. Ranson *et al*. [34] recently published a country wide study and showed that *An. arabiensis* populations from Sudan are resistant to both DDT and pyrethroids, but remained fully susceptible to carbamates and the organophosphate, fenitrothion. This supports what was observed in the SENN-DDT colony.

The fact that DDT and pyrethroid resistance in *An. gambiae* are linked has been well-documented and has been attributed to the presence of *kdr* mutations [23,25]. Specifically, *kdr* is strongly linked with DDT and permethrin resistance, and less so with deltamethrin resistance [27,40]. In *An. arabiensis,* the relationship between the presence of *kdr* mutations and resistance phenotype is also complicated [2,41]. The SENN-DDT colony is fixed for the L1014F mutation. The South African *An. arabiensis* population has previously been confirmed not to carry any *kdr* mutations [7,8]. However, the continued selection pressure from exposure of MBN-DDT to DDT has resulted in this colony being fixed for the L1014F mutation. The L1014S mutation is absent from both laboratory colonies.

The detoxification enzyme profiles of the two laboratory selected DDT-resistant *An. arabiensis* strains was investigated using cross-species hybridizations of *An. arabiesnsis* genetic material with the *An. gambiae* detoxification microarray (detox chip). Of the 98% of probes that hybridized, only one gene in the SENN-DDT colony was over-transcribed. This was a cytochrome P450, *CYP9L1.* This was in contrast to the MBN colony where a similar success rate of probe hybridization was recorded, but 20 genes were highly transcribed in the resistant phenotype.

The use of the *An. gambiae* detox chip allows for the evaluation of transcription of a large number of genes simultaneously, but the criteria one uses to find significance will determine how many genes are of interest for further study. In other studies (both same- and cross-species hybridizations) the cut-off for significance in terms of fold change ranged from >1.5 to 2.0, and the *p*-value cut-off for significance ranged form < 0.001 to <0.05 [32,33,42-44]. Generally, where a higher fold-change was used as criteria to identify over-transcribed genes, a lower *p*-value cut-off was also used to determine significance, and vice versa. In this study, the stringency was adjusted for the wash solutions by increasing the required amount of each solution (i.e. higher than what was recommended by the supplier). The experimental conditions selected produced the best arrays, but because the experiment was based on cross-species hybridizations, we chose to use less strict criteria for identifying those genes with a significant level of differential transcription.

The action of the P450-dependent monooxygenases is one of the ways in which insects become resistant to insecticides [16]. Only one gene, *CYP9L1,* showed high expression levels in the SENN resistant phenotype and is likely to play a key role in the observed resistance to deltamethrin. The CYP9 gene family is closely related to the CYP6 family (highly expressed in the MBN resistant phenotype) [45] and members have been linked to insecticide resistance in a number of insects [44-46]. Although not likely to be the case here, it is interesting to note that a single P450 enzyme has been implicated in resistance to DDT [47,48].

Five genes were consistently saturated when both MBN- and SENN-DDT arrays were analysed. Some of these were mainly saturated in one channel, and less so in the other, which raises the possibility that a gene is over-transcribed, but this is masked by the saturation, and might therefore be overlooked. Two of these, *CYP4G16* and *CO1,* were investigated further using qPCR and SENN-DDT genetic material. The monoxygenase, *CYP4G16* was chosen because it has previously been linked to pyrethroid tolerance in *An. arabiesnis*[31]. The cytochrome oxidase gene, *CO1,* was selected as it was over-transcribed in a microarray study on pyrethroid resistant *An. funestus*[32]. In this study, we obtained FC values of 1.8 and 1.6 for *CYP4G16* and *CO1* respectively after qPCR analysis. While these values are relatively low when compared with previously reported data, their involvement, if any, in resistance and the reason for saturation on the microarrays should be investigated further.

According to our criteria, 20 genes were differentially regulated in the resistant MBN colony and most of these genes belong to the monooxygenase and GST enzyme groups. In addition, most of the over-transcribed CYP genes belonged to the CYP6 family, which is frequently associated with insecticide resistance in insects. The top four genes were selected for qPCR validation. These were, in order of significance, *CYP6M2, TPX4, CYP6AK1* and *CYP6P3.* Recently, Munhenga and Koekemoer [49] used qPCR to assess the transcription of a range of monooxygenase genes in a pyrethroid-selected *An. arabiensis* colony from the same geographical area (KZN, South Africa). They found that *CYP6Z1* (FC = 4.7), *CYP6Z2* (FC = 1.7) and *CYP6M2* (FC = 2.2) were significantly over-transcribed. Interestingly, in our evaluation of *CYP6M2,* qPCR produced a FC of 2.2, the same level as that reported by Munhenga and Koekemoer [49], even though a different reference gene was used between the two studies.

Of the CYP genes that were over-transcribed in this study according to microarray evaluation, a number have been implicated in insecticide resistance in *An. gambiae.* Djouaka *et al*. [50] found that *CYP6P3* and *CYP6M2* were both upregulated in pyrethroid-resistant An. gambiae populations in Benin and Southern Nigeria. In permethrin-resistant *An. gambiae* from Ghana, *CYPM2CYP6AK1* and *CYP6P3* were amongst the top 10 differentially expressed genes in resistant mosquitoes [30]. The authors found that the outcomes of the microarray and qPCR data were similar as was confirmed in the present study.

The GSTs also featured prominently in the enzyme profile of resistant MBN colony. The epsilon class GSTs have been specifically linked to DDT resistance in *An. gambiae*[29,51-54] and delta class GSTs to a lesser extent [52]. Furthermore, GSTs have more recently been linked to pyrethroid resistance in other insects [55,56] and so their presence in the resistance profile of MBN-DDT might be linked directly to protection against the pyrethroid, deltamethrin. Because they help to protect cells against oxidative stress, their over-expression in the MBN-DDT colony is also likely to be linked to the action of the cytochrome P450s where the GSTs are involved in secondary metabolism through the action of glutathione peroxidase [52].

A number of enzymes, namely the SODs, TPXs and GRXs, counteract the effects of reactive oxygen molecules, which are harmful to the host [57]. The SODs function by converting superoxide anions to hydrogen peroxide and oxygen [58]. In turn, the TPXs are involved in the removal of hydrogen peroxide [58]. Based on microarray experiments, we reported high levels of *TPX4* (2.3 fold) expression in the South African population of DDT selected *An. arabiensis*. This enzyme was over-transcribed in *An. arabiensis* during the spraying season of a cotton field in Cameroon [31], while *TPX1* was over-expressed in *An. gambiae,* resistant to pyrethroids, from Ghana [59]. In the MBN colony, whether the high expression of *TPX4* is related directly to the activities of the P450 enzymes (to counteract metabolic byproducts), or is a function of the insecticide resistance selection process where they are on “stand-by” to provide protection against pyrethroids, is unknown.

According to Brooke and Koekemoer [27], and references therein, the correlation between the presence of *kdr* and mosquito response to insecticide is strongest in the case of DDT, less so with permethrin, and weakest with deltamethrin. The outcome of the synergist studies performed here suggests that detoxification enzymes have no impact on DDT resistance in these strains, but are very important for protection against the pyrethroid, deltamethrin. The presence of the L1014F *kdr* mutations is likely to assist in protection against permethrin.

## Conclusions

The combination of expression data and synergist data suggests that the systems in place for insecticide resistance are extremely complex. There is a lack of understanding as to how these genes interact and support each other in the detoxification of specific insecticides and further investigation into these molecular mechanisms is needed. It is clear that the metabolic genes associated with each resistant colony are unique for that population and there was no single gene that showed an increase in transcription between South Africa and Sudan. However, a number of genes identified in this study as being over-transcribed have been flagged in other studies for their possible roles in insecticide resistance of *An. arabiensis.* It would be valuable to replicate this study in wild populations from these regions and compare the results of enzyme studies based on laboratory colonies and wild-caught mosquitoes.

## Abbreviations

WHO, World Health Organization; DDT, Dichlorodiphenyltrichloroethane; GST, Glutathione S-transferase; P450, Cytochrome oxidase/P450; SOD, Superoxide dismutase; TPX, Thioredoxin peroxidase; kdr, Knockdown resistance; IRS, Indoor residual spraying; LLIN, Long-lasting insecticide treated bednet; KZN, KwaZulu-Natal.

## Competing interests

The authors declare that they have no competing interests.

## Authors’ contributions

LN conducted the experiments and data analyses, interpreted results, and drafted the first version of the manuscript. RC provided technical support for the duration of the study, particularly with regard to the microarrays and qPCR, and contributed to the editing of the manuscript. NC participated in the microarray data analysis and provided useful comments for the manuscript. HR contributed to revision of the manuscript. MC provided funding for the study and contributed to revision of the manuscript. LLK provided funding for the study, conceived the project, participated in coordinating the study and helped with revision of the manuscript. All authors have read and approved the final manuscript.
